# Disulfide by Design 2.0: a web-based tool for disulfide engineering in proteins

**DOI:** 10.1186/1471-2105-14-346

**Published:** 2013-12-01

**Authors:** Douglas B Craig, Alan A Dombkowski

**Affiliations:** 1 Department of Pediatrics, Wayne State University School of Medicine, Detroit, Michigan 48201, USA

**Keywords:** Disulfide bond, Protein design, Protein engineering, Bioinformatics

## Abstract

**Background:**

Disulfide engineering is an important biotechnological tool that has advanced a wide range of research. The introduction of novel disulfide bonds into proteins has been used extensively to improve protein stability, modify functional characteristics, and to assist in the study of protein dynamics. Successful use of this technology is greatly enhanced by software that can predict pairs of residues that will likely form a disulfide bond if mutated to cysteines.

**Results:**

We had previously developed and distributed software for this purpose: Disulfide by Design (DbD). The original DbD program has been widely used; however, it has a number of limitations including a Windows platform dependency. Here, we introduce Disulfide by Design 2.0 (DbD2), a web-based, platform-independent application that significantly extends functionality, visualization, and analysis capabilities beyond the original program. Among the enhancements to the software is the ability to analyze the B-factor of protein regions involved in predicted disulfide bonds. Importantly, this feature facilitates the identification of potential disulfides that are not only likely to form but are also expected to provide improved thermal stability to the protein.

**Conclusions:**

DbD2 provides platform-independent access and significantly extends the original functionality of DbD. A web server hosting DbD2 is provided at http://cptweb.cpt.wayne.edu/DbD2/.

## Background

Disulfide bonds provide stability to many extracellular and secreted proteins. Disulfide bonds are believed to decrease the conformational entropy and raise the free energy of the denatured state, thus providing an increase in stability to the folded protein conformation [1]. While the overall effect of a disulfide bond may be complex, including an enthalpic component [2,3], considerable evidence supports the long-standing hypothesis that stability is gained through a reduction in unfolded conformational entropy [4,5]. Many studies have sought to utilize engineered disulfide bonds to increase the stability of proteins in biomedical and industrial applications. Interestingly, not all engineered disulfides have provided an increase in stability, as there are a number of reports of destabilizing disulfides. Given the mixed outcomes, disulfide engineering studies would benefit greatly from computational tools that not only identify novel disulfides that are likely to form, but also indicate whether a disulfide is likely to confer an increase in stability.

To investigate factors that may explain why some engineered disulfides are stabilizing while others destabilize the protein, one report summarized structural features from previous studies of engineered disulfides where stability data and crystal structures were available [6]. Supporting theoretical models that propose a stabilizing effect due to a reduction in unfolded state conformational entropy, the authors found that a large fraction of the stabilizing mutations were associated with longer loop lengths (between 25 and 75 residues) bridged by the disulfide bond, while few stabilizing mutations were reported for shorter loop lengths (<25 residues). The authors also determined that stabilizing disulfides were predominantly found spanning regions of relatively high mobility as assessed by the residue B-factors. The B-factor (temperature factor) is a measure of dynamic mobility for each atom. These conclusions are consistent with the seminal experiments of the Matthews group using T4 lysozyme. Their disulfide engineering experiments more than 20 years ago found that large loop lengths and high B-factors are conducive to stabilizing disulfide bonds [7]. A recent study utilized B-factors to help select potential mutations for disulfide engineering with the goal of increasing the thermal stability of *Candida antarctica* lipase B, a widely used industrial enzyme [8]. From a lengthy list of potential mutations predicted by two disulfide engineering algorithms, the authors further ranked the candidate disulfide bonds using the B-factors of the residue pair associated with each bond. For each potential disulfide, the B-factors for the associated pair of residues were summed and the candidate disulfides ranked accordingly, from highest mobility to lowest. The four candidate disulfides having the greatest B-factor were selected for mutation and subsequent thermal stability analysis, along with one lower ranked candidate. The disulfide bond that provided the greatest improvement in thermal stability was the candidate having the highest B-factor. The authors found that the change in thermal stability associated with each novel disulfide bond was correlated to the change in mobility of the mutated residue pair. Other recent studies support the rationale of improving thermal stability of proteins by disulfide bonding of regions having high flexibility [9,10]. Given the cumulative evidence demonstrating high mobility regions as favorable to engineered disulfides that improve thermal stability, we have added features in DbD2 that enable B-factor analysis of protein regions involved in candidate disulfide bonds.

The design of novel disulfide bonds in a protein involves the use of a structural model to identify residue pairs that can be mutated to cysteines to form the novel bond. Although the selection of candidate pairs can sometimes be performed simply based on proximity alone, successful disulfide engineering is greatly facilitated by consideration of the strict geometric constraints necessary for the introduction of a disulfide. A number of computational methods have been developed for the prediction of protein sites suitable for disulfide formation, dating back to the work of Pabo and Suchanek [11-14]. These methods generally follow a similar modeling paradigm based on bond geometry found in native disulfide bonds. Our original disulfide engineering algorithm, Disulfide by Design, utilized native geometry and was based on methods developed for protein fold recognition [15,16]. One advantage of our software is that it calculates an energy value for each candidate disulfide, thus providing a means to rank potential disulfide bonds. The original Disulfide by Design application has been downloaded over 1000 times and used in a wide variety of applications [17-21]. While it has proven very useful in numerous disulfide engineering projects, our original software has a number of limitations. The application was originally compiled with Windows-specific dependencies, and it also requires local installation. Additionally, the software is limited in the size of proteins it can analyze (5000 residues), and it is unable to accommodate multiple structural models, for example those often associated with NMR structures. We have rewritten the original Windows-based program to overcome these limitations and to implement additional enhancements. The redesigned program includes a number of important analysis features as described below. Disulfide by Design 2.0 is freely available for non-profit use at: http://cptweb.cpt.wayne.edu/DbD2/.

## Implementation

Disulfide by Design 2.0 re-implements the original design algorithm, adds numerous enhancements, and provides a web-based interface. The application can be accessed through any web-browser, and is therefore platform-independent. This implementation also ensures that updates, improvements, and bug-fixes are immediately available to the user without the need to reinstall application software on their computer. A complete history of software version updates is maintained online as part of the web application. In addition to all the functionality found in the standalone version, the new web-based version significantly improves and extends both functionality and visualization.

While still allowing protein structure files to be loaded from the local desktop, DbD2 now provides direct import of files from the Protein Data Bank (PDB) simply by specifying the PDB identifier. There is a security limit of 2 MB imposed on the size of local files which may be uploaded to the server, but there is no size limit for files retrieved directly from the PDB website. As with the previous version, there are several user adjustable parameters regarding the stringency of disulfide bond geometric requirements, and the application generates a list of candidate residue pairs meeting the specified geometric constraints. Structures are analyzed incrementally with a percent-complete estimate displayed during analysis. For PDB files containing multiple models (e.g., those generated by NMR), the user is now given a list of all available models from which to choose from, overcoming a limitation of the original software. Analysis can be performed on one selected model at a time, results can be saved between runs, and 3D visualization across models is possible. Disulfide by Design 2.0 now includes the ability to visualize protein models and potential bond sites in both two and three dimensions. Once loaded, a detailed graphic schematic of the protein secondary structure is available to the user.

The application now includes four tabbed pages: file information, analysis, 2° structure, and 3-D view. The file information tab provides a summary of structural information and a scrollable page of the entire PDB file. The analysis tab displays the residue pairs that meet the geometric requirements for disulfide bond formation if mutated to cysteines (Additional file 1: Figure S1). The disulfide bond energy and B-factor are listed for each potential disulfide. The B-factor is calculated for each residue pair by summing the values for the two residues, each representing the average B-factor of the backbone and β-carbon atoms. The range and mean B-factor are displayed on the file information tab. These values are provided as guidance when selecting potential disulfides based on B-factors. The analysis output can be sorted on any field, allowing for quick ranking of candidate disulfides. Any number of potential disulfides can be selected via check boxes for subsequent analysis on the 2° structure and 3-D view tabs. The 2° structure tab is entirely new to DbD2 (Additional file 2: Figure S2). It provides a linear representation of the protein secondary structure and flags the locations associated with potential and selected disulfides identified on the analysis tab. Below the secondary structure representation a linear colorimetric bar spans the length of the protein chain, and the color at each residue position represents the B-factor value. The displayed B-factor color is normalized to the minimum and maximum values found in the given protein, with red indicating a high B-factor (high mobility) and blue representing a low value. The color scale is normalized to 512 discrete values between the minimum and maximum B-factor values for all residues in the protein. Each residue B-factor is color coded on a 256-step blue scale for the lower half and on a 256-step red scale for the upper half. This feature allows the user to easily assess the relative B-factor for locations of potential disulfides. Moving the mouse over individual residue positions provides the raw B-factor value derived from the PDB file, residue identity, and detailed secondary structure information. Additionally, predicted disulfide connectivity is provided for the residue. Additional file 2: Figure S2 shows an example from PDB structure 1TCA, where mouse-over of residue 308 reveals predicted disulfide bonding to residue 162.

The new 3-D view tab provides a fully integrated molecular viewer with dual windows, enabling the simultaneous display of native and mutant protein structures (Additional file 3: Figure S3). We utilized the open-source Jmol molecular viewer (http://www.jmol.org/), which offers extensive options for viewing and structural manipulation. Disulfide bonds selected on the analysis tab followed by a click on the “create/view mutant” button are displayed in the mutant structure panel. Convenience buttons are provided for toggling between cartoon and wireframe renderings as well as for hiding/showing disulfides. The wild type and mutant structures can be rotated, magnified, and manipulated independently. Additionally, a very helpful feature in DbD2 facilitates comparison of the two structures. The orientation and perspective of either view is instantly copied to the other view by simply pressing the “copy orientation” button. If multiple models are available in a single PDB file, then it becomes possible to view and compare two different models side-by-side.

**Figure 1:**
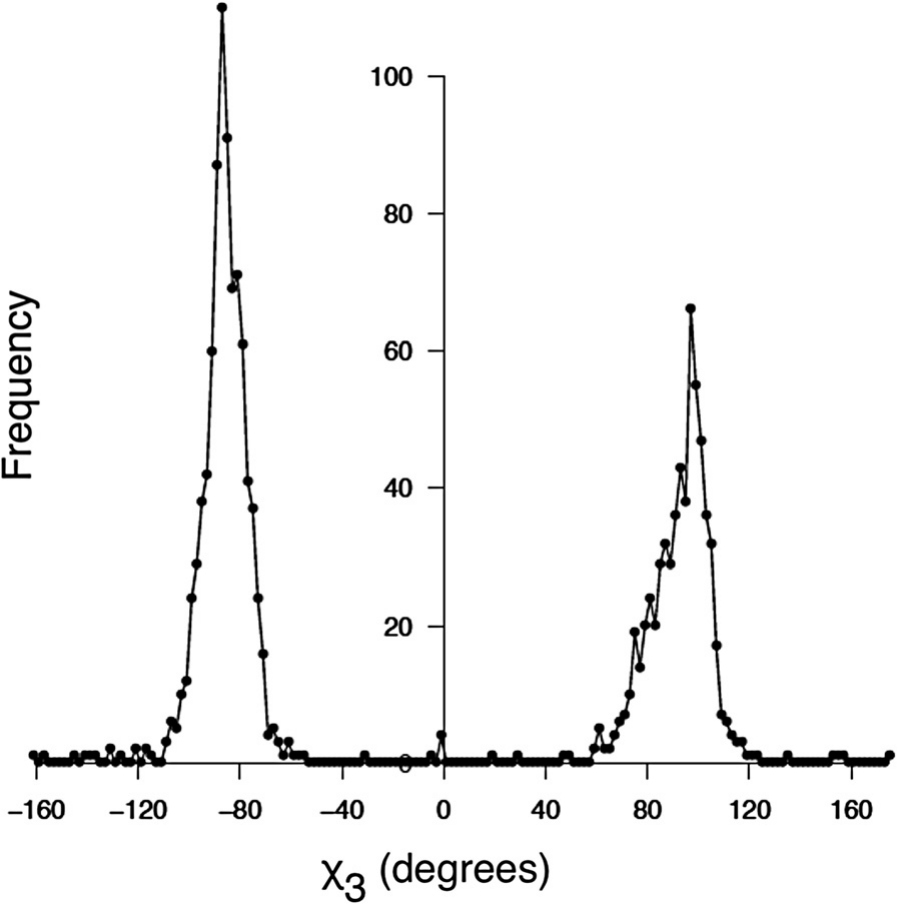
**Distribution of *χ***_**3 **_**torsion angles observed in 1505 native disulfide bonds found in 331 PDB protein structures.** Peaks occur at -87 and +97 degrees.

**Figure 2:**
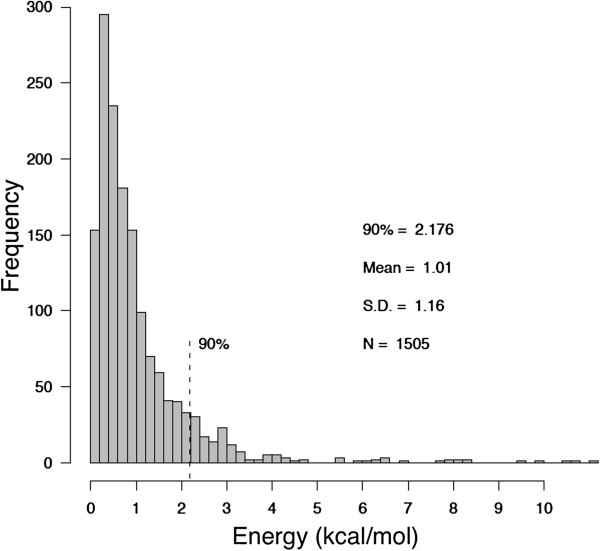
**Distribution of the disulfide bond energy calculated for 1505 native disulfide bonds in our survey set using the DbD2 energy function.** The mean value is 1.0 kcal/mol, and the 90th percentile is 2.2 kcal/mol.

Other enhancements include: the ability to save all results in standard comma-separated (CSV) format for use in standard spreadsheet programs, and the ability to export mutant PDB files for subsequent use in molecular dynamics simulations or import to other molecular modeling packages. We have also updated our energy function to reflect torsion angles observed in a large number of native disulfide bonds. Our original energy minima, as defined in equations 1–4 of [15], were based on values published in an early survey of native disulfide bonds [22]. That study utilized 351 disulfide bonds found in 131 protein structures, and a distribution of χ_3_ torsion angles observed in the survey set revealed peaks at -80 and +100 degrees. The χ_3_ torsion angle is defined by rotation of the two C_β_ atoms about the S_γ_-S_γ_ bond. In this work we analyzed disulfide geometric characteristics in an expanded set of PDB structures and found slightly different values. Using 1505 native disulfide bonds in 331 non-homologous proteins, we observed χ_3_ peaks at -87 and +97 degrees (Figure 1). We have updated our energy function to provide χ_3_ torsion angle minima at these values, replacing equation (3) of [15] with *E*(*χ*_3_) = 4.0[1 - *cos*(1.957[*χ*_3_ + 87])], where χ_3_ is in degrees. Figure 2 shows the distribution of energy values in the 1505 native disulfide bonds using our updated function. We found that 90% of native disulfides have an energy value less than 2.2 kcal/mol. This value provides a convenient guideline when considering potential disulfide bonds with DbD2. The energy parameter provides a relative value that can be compared between candidate disulfides to identify potential bonds that best conform to native disulfide geometry.

## Results and discussion

**Figure 3:**
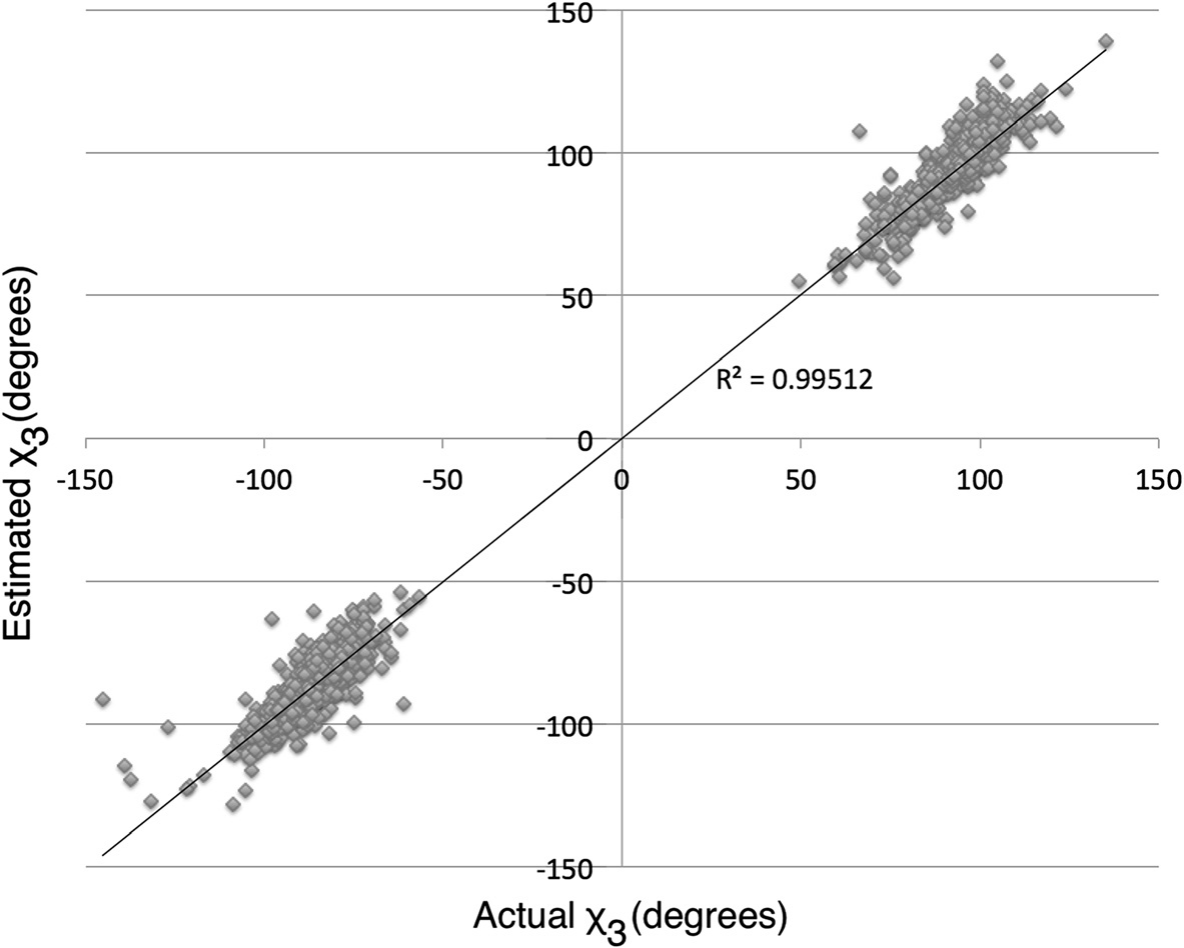
**Correlation of the estimated and actual *χ***_**3 **_**torsion angles for the 1418 native disulfide bonds that were correctly identified with DbD2 and also were predicted to have the correct chirality.** An R^2^ value of 0.995 demonstrates accurate prediction of disulfide atomic coordinates.

Validation of DbD2 was performed with a blind test, predicting potential disulfide bonds in the aforementioned 331 non-homologous PDB structures. These structures were selected with the criteria of: 1) having at least one disulfide bond; 2) less than 50% sequence identity; and 3) structural resolution ≤2.0 angstroms. We did not split our set of proteins into independent training and test sets because: 1) the DbD2 algorithm uses only the coordinates of the backbone and C_β_ carbon atoms for bond predictions; therefore, the side chain identities and locations of native disulfides were hidden in the test; and 2) it is preferable to use the largest possible set of disulfides for training the model as evidenced by the energy function improvements implemented in this release based on the expanded training set. DbD2 was used with default settings to predict suitable locations for disulfide bonds. Of the 1505 native disulfide locations, the algorithm correctly predicted 1479 (98%) as appropriate for disulfide formation. Of the 1479, chirality of the χ_3_ torsion angle was correctly predicted in 1418 (96%) of the bonds. In these cases, a very strong linear correlation is observed between the predicted and true χ_3_ angles, with an R^2^ value of 0.995 (Figure 3). Furthermore, the modeled positions of the sulfur atoms involved in the predicted disulfide bonds were exceptionally accurate. The median distance of the predicted sulfur location from the actual location found in the PDB structure was 0.08 angstroms.

**Figure 4:**
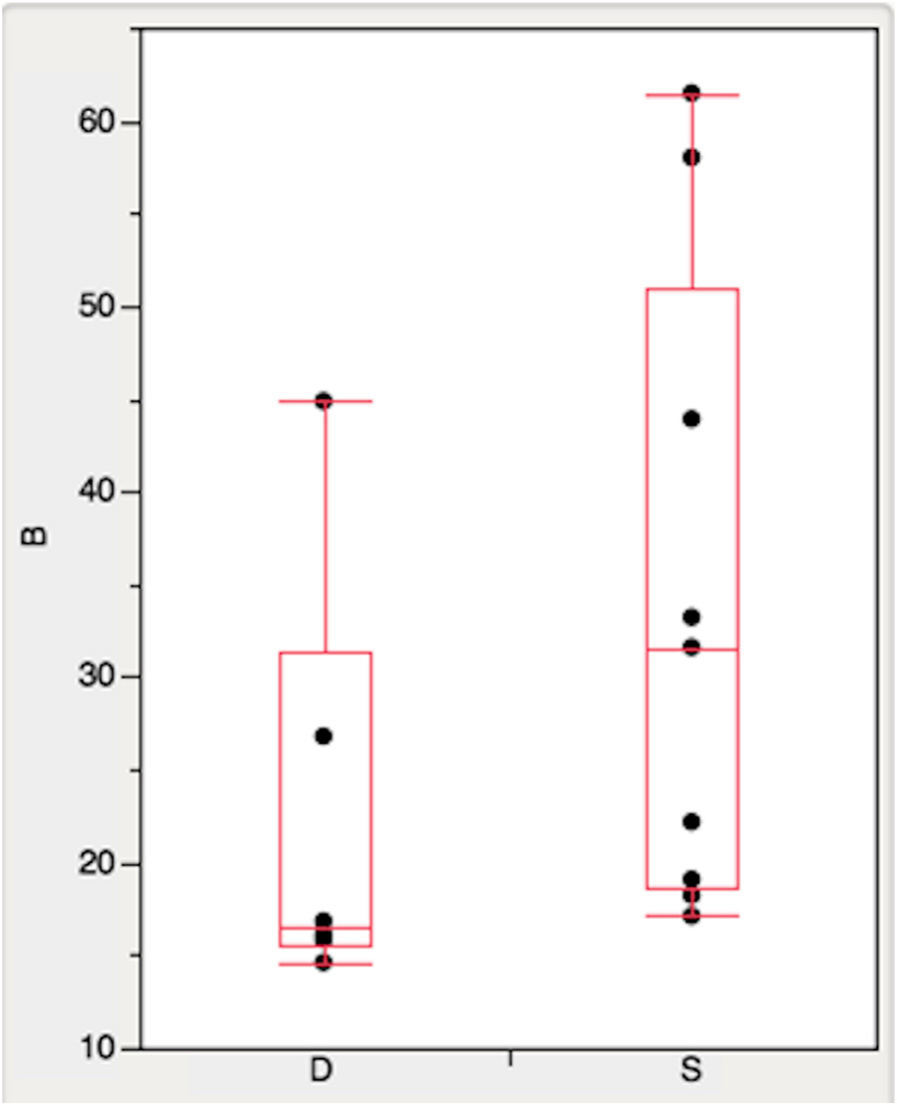
**Comparison of native residue B-factors in stabilizing and destabilizing engineered disulfide bonds.** The native structures associated with engineered disulfides previously reported as stabilizing (S) or destabilizing (D), based on experimental evidence, were analyzed with DbD2. The mean B-factor for residues involved in stabilizing disulfide bonds was 31.6 compared with 16.5 for those involved in destabilizing bonds, *P* = 0.066.

The results tab provides the ability to sort potential disulfides using any of the displayed parameters by simply clicking on the column header. We investigated parameter ranking methods to assist the user in selecting disulfides expected to stabilize a protein. We utilized the same set of engineered disulfides previously summarized in Table I of Dani et al. that were categorized as either stabilizing or destabilizing based on experimental evidence [6]. We used DbD2 to perform predictions for each of the engineered disulfide locations using the wild type structure. The DbD2 algorithm did not predict approximately half of the engineered disulfides, primarily because they exceeded the allowed bond angles or lengths of the model. This result is similar to that reported by Dani et al. with the MODIP algorithm. For the disulfides with successful predictions we compared the DbD2 energy and ΣB-factor parameters between stabilizing and destabilizing disulfides. We found no significant difference in energy value between the two categories (data not shown). However, the ΣB-factor was considerably higher for the stabilizing disulfides as compared to destabilizing bonds, with mean values of 31.6 and 16.5 respectively (Figure 4). The statistical significance calculated with the Mann–Whitney U test (two tailed) was *P* = 0.066. Dani et al. had also reported that engineered disulfides that increase stability are associated with protein regions having higher B-factors. We found that ranking by the ΣB-factor alone is a better predictor of stabilization than when using a score derived from a combination of equally weighted energy and B-factors ranks. It is important to note that B-factors vary widely between protein structures due to the refinement procedure, resolution, and crystal contacts [23-25]. Therefore, when considering potential stabilizing disulfides it is preferable to compare residue B-factors relative to the target protein structure. The colorimetric B-factor scale available in the secondary structure tab of DbD2 is intended to facilitate this analysis.

Another structural property previously associated with the stabilizing effect of engineered disulfides is residue depth. It was reported that stabilizing disulfides are preferentially located close to the protein surface [6]. However, the observation that both B-factor and residue depth are determinants of the stability imparted by a disulfide bond likely reflects the dependency between residue burial and residue flexibility. An early study of 110 protein structures found a bimodal distribution of normalized B-factors [23]. The low B-factor peak reflected buried residues, while the high B-factor peak was associated with surface exposed residues. More recent reports have also reported a correlation between flexibility (B-factor) and residue depth [24]. One study found a strong linear correlation between the normalized B-value and the distance of the residue from the protein surface [25]. To avoid using correlated parameters in predicting the stabilizing effect of a disulfide bond we focused on the term that directly reflects flexibility (i.e., B-factor).

The above results highlight the difference between engineered disulfides that are likely to form and those expected to improve stability. For the former group, we believe that the energy value provides the preferable method to rank putative disulfides as it reflects how well the modeled bond conforms to known disulfide geometry. The effect of a given disulfide on the overall stability of a protein appears to be dependent on multiple factors. Based on previous reports we have implemented B-factor analysis in DbD2 to assist in the identification of potential disulfides that may confer an improvement in stability. As demonstrated by Le et al., a reasonable strategy for the identification of novel disulfides that improve thermal stability is to first identify putative disulfides that have energy values consistent with native disulfides (Figure 2) and then rank the candidates by the ΣB-factor parameter [8]. As we expand our understanding of the biophysical properties that dictate the effect of a disulfide on the stability of a protein we will be able to improve predictive algorithms. Recent reports suggest that a range of factors, including kinetic effects, warrant consideration [26].

## Conclusions

In this work we have updated and enhanced our previous Windows-based program, Disulfide by Design, to create a full-function web-based application, DbD2. This extends availability of the application to non-Windows users, and eliminates the need to install and update the program on individual user machines. In addition to making DbD2 platform independent, we have significantly updated the previous version by adding numerous features to support disulfide engineering, including visualization tools and consideration of structural mobility at locations of potential disulfides. Previous reports have established these locations as favorable for engineered disulfides that improve thermal stability.

## Availability and requirements

**Project name:** Disulfide by Design 2.0

**Project home page:**http://cptweb.cpt.wayne.edu/DbD2/

**Operating system(s):** Platform independent

**Programming language:** Python / PHP

**Other requirements:** none

**License:** see home page

**Any restrictions to use by non-academics:** license necessary for commercial use

## Competing interests

The authors declare that they have no competing interests.

## Authors’ contributions

DC and AD contributed to the software design and testing. DC implemented the software. Both authors drafted, edited, and approved the final manuscript. Both authors read and approved the final manuscript.

## Supplementary Material

Addditional file 1: Figure S1 The analysis tab shows disulfide design parameters, predicted disulfide bonds, and allows selection of bonds for display in the secondary structure and 3-D tabs. Click here for file

Addditional file 2: Figure S2 The secondary structure tab shows the relationship between the linear protein chain, secondary structure, and predicted disulfide bonds. Residues associated with potential disulfides are highlighted in gold, while selected bonds are shown in red. Mouse-over of residue positions provide detailed residue information including raw B-factor and predicted disulfide connectivity. Normalized B-factors are represented on a colorimetric bar below the secondary structure. Red indicates high B-factor values while blue represents low values. Click here for file

Addditional file 3: Figure S3 The 3-D view tab provides a fully interactive structural viewer that displays disulfide bonds selected in the analysis tab. Dual windows allow simultaneous display of wild type and mutant protein structures. The perspective of the two views can be easily synched with the “copy orientation” button. Click here for file
