# Supplementary figures and images for: Disulfide by Design 2.0: a web-based tool for disulfide engineering in proteins
Source: BMC Bioinformatics. 2013 Dec 1;14:346. doi: 10.1186/1471-2105-14-346 (PMC3898251; doi:10.1186/1471-2105-14-346)

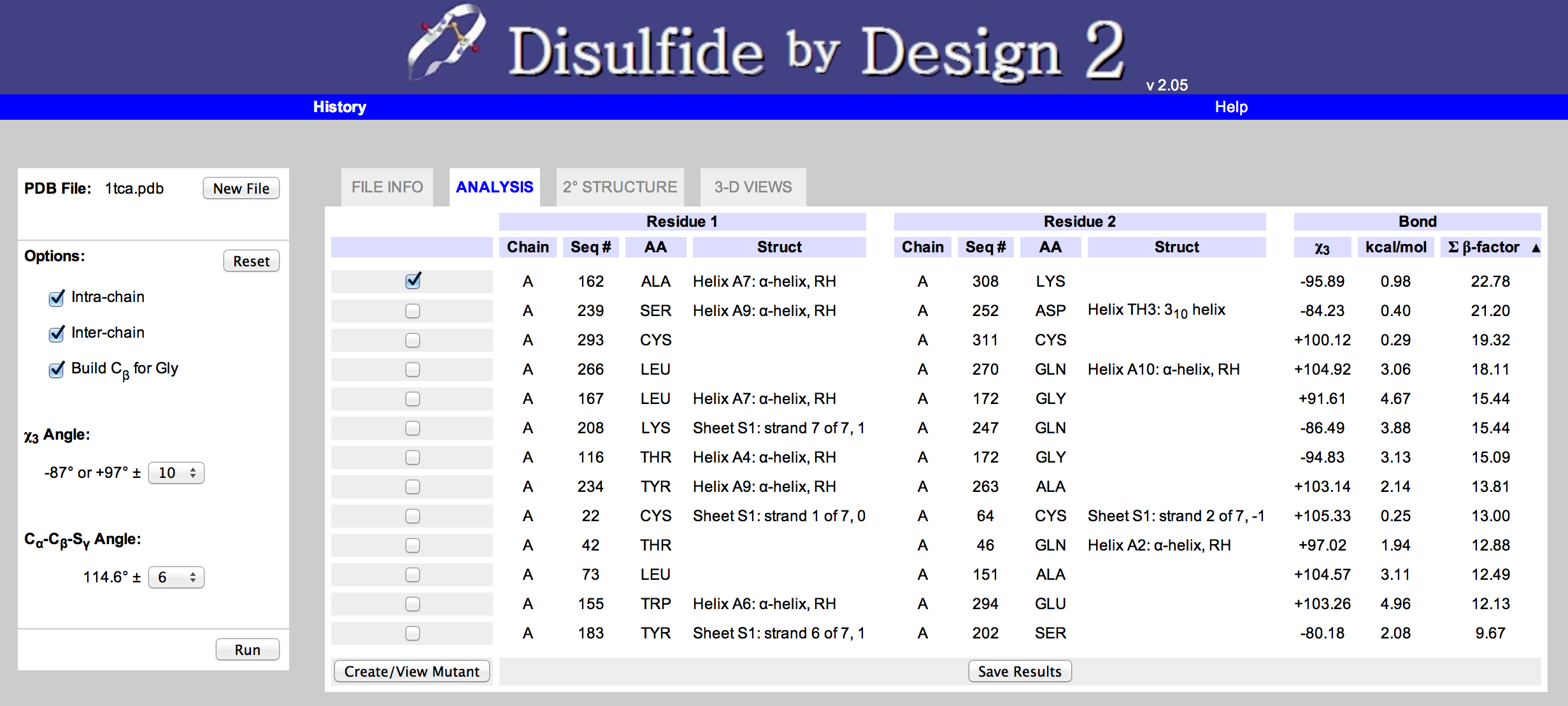

Supplement: Addditional file 1: Figure S1 — The analysis tab shows disulfide design parameters, predicted disulfide bonds, and allows selection of bonds for display in the secondary structure and 3-D tabs. [file 1471-2105-14-346-S1.png]

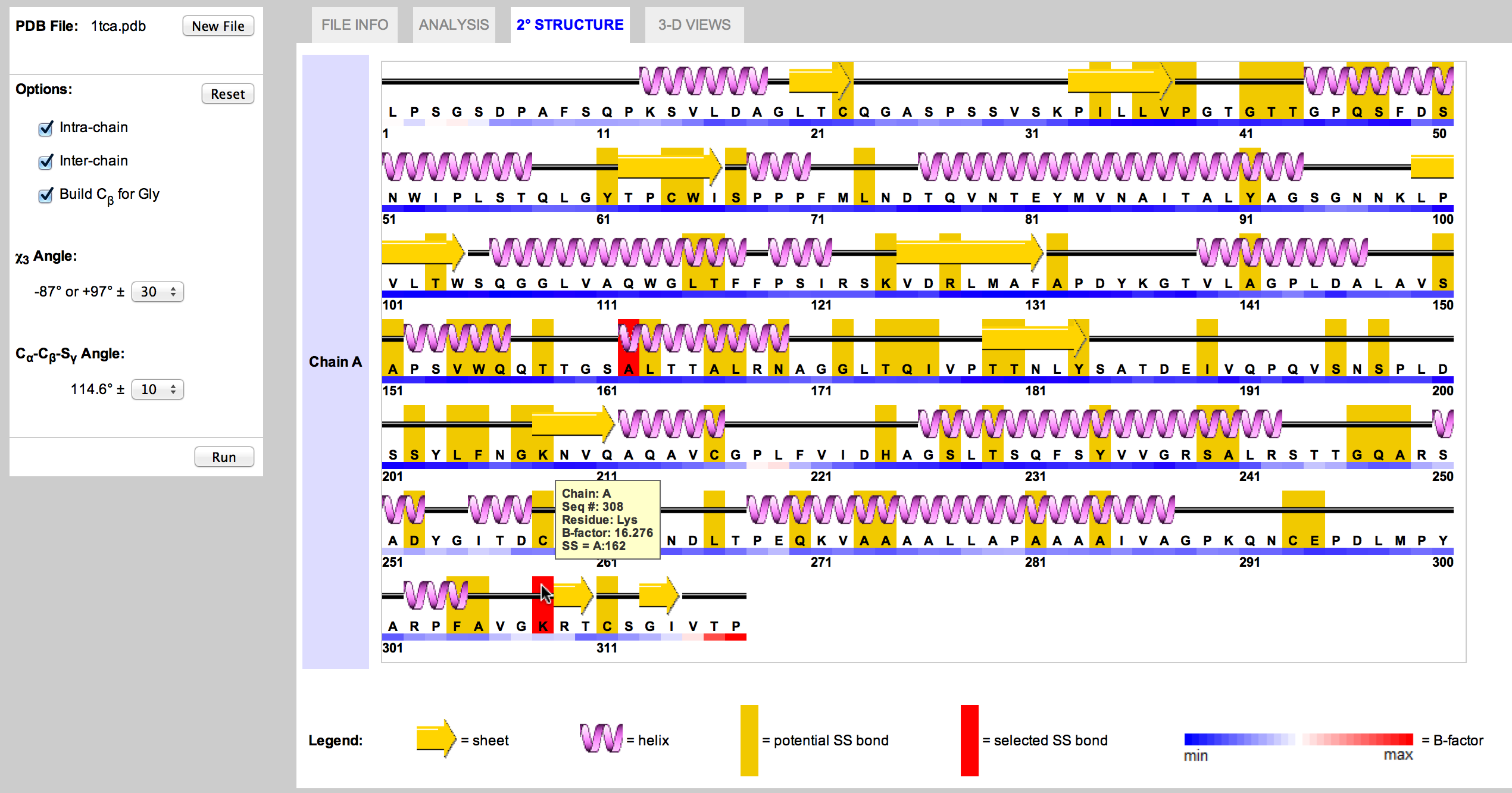

Supplement: Addditional file 2: Figure S2 — The secondary structure tab shows the relationship between the linear protein chain, secondary structure, and predicted disulfide bonds. Residues associated with potential disulfides are highlighted in gold, while selected bonds are shown in red. Mouse-over of residue positions provide detailed residue information including raw B-factor and predicted disulfide connectivity. Normalized B-factors are represented on a colorimetric bar below the secondary structure. Red indicates high B-factor values while blue represents low values. [file 1471-2105-14-346-S2.png]

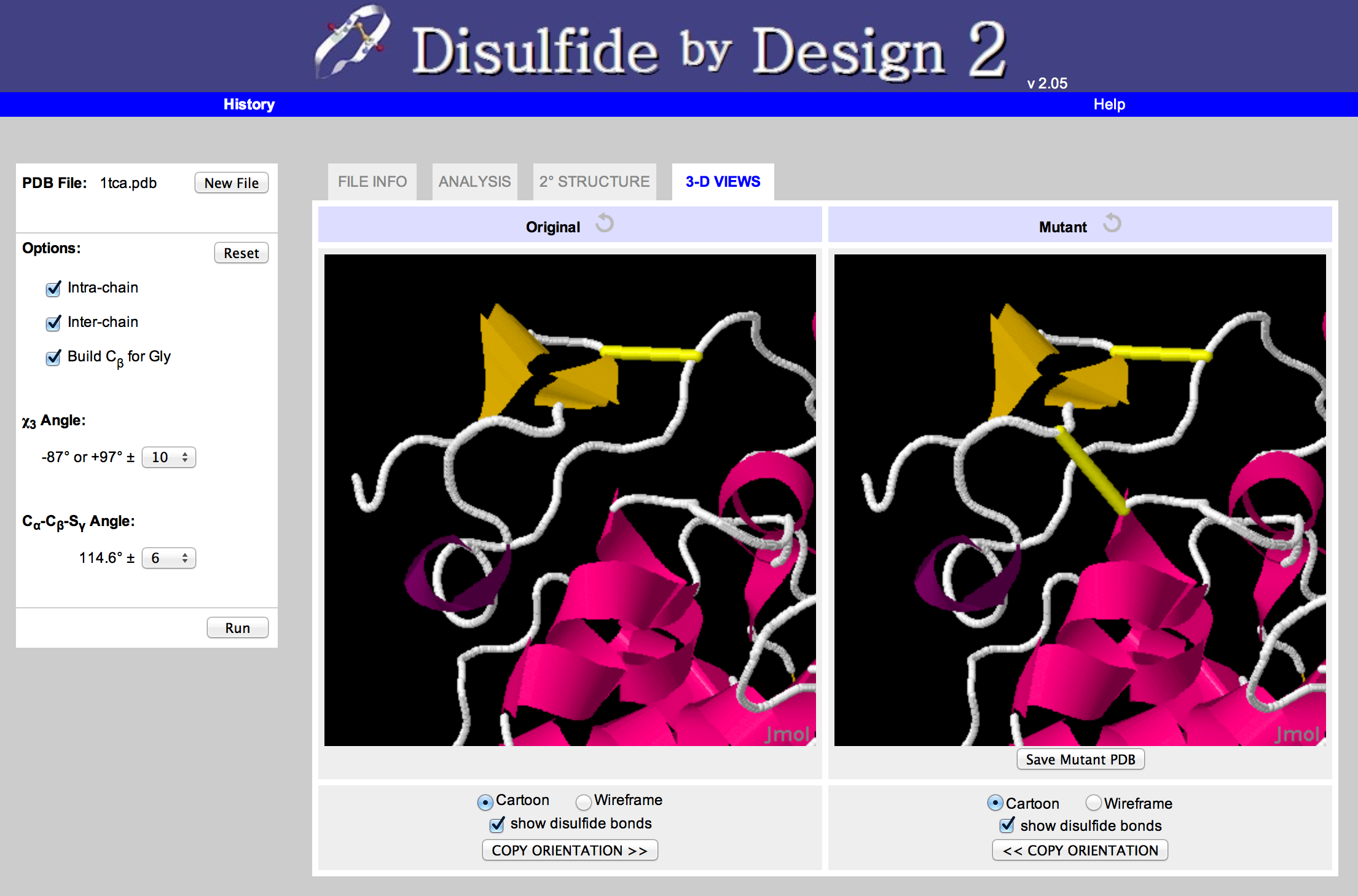

Supplement: Addditional file 3: Figure S3 — The 3-D view tab provides a fully interactive structural viewer that displays disulfide bonds selected in the analysis tab. Dual windows allow simultaneous display of wild type and mutant protein structures. The perspective of the two views can be easily synched with the “copy orientation” button. [file 1471-2105-14-346-S3.png]
